# Fabrication of Nanostructured Supramolecules through Helical Inclusion of Amylose toward Hydrophobic Polyester Guests, Biomimetically through Vine-Twining Polymerization Process

**DOI:** 10.3390/biomimetics8070516

**Published:** 2023-11-01

**Authors:** Jun-ichi Kadokawa

**Affiliations:** Graduate School of Science and Engineering, Kagoshima University, 1-21-40 Korimoto, Kagoshima 890-0065, Japan; kadokawa@eng.kagoshima-u.ac.jp; Tel.: +81-99-285-7743

**Keywords:** amylose, enzymatic polymerization, glucan phosphorylase, inclusion complex, hydrophobic polyester, nanostructure, supramolecular network, vine-twining polymerization

## Abstract

This review article presents the biomimetic helical inclusion of amylose toward hydrophobic polyesters as guests through a vine-twining polymerization process, which has been performed in the glucan phosphorylase (GP)-catalyzed enzymatic polymerization field to fabricate supramolecules and other nanostructured materials. Amylose, which is a representative abundant glucose polymer (polysaccharide) with left-handed helical conformation, is well known to include a number of hydrophobic guest molecules with suitable geometry and size in its cavity to construct helical inclusion complexes. Pure amylose is prepared through enzymatic polymerization of α-d-glucose 1-phosphate as a monomer using a maltooligosaccharide as a primer, catalyzed by GP. It is reported that the elongated amylosic chain at the nonreducing end in enzymatic polymerization twines around guest polymers with suitable structures and moderate hydrophobicity, which is dispersed in aqueous polymerization media, to form amylosic nanostructured inclusion complexes. As the image of this system is similar to how vines of a plant grow around a support rod, this polymerization has been named ‘vine-twining polymerization’. In particular, the helical inclusion behavior of the enzymatically produced amylose toward hydrophobic polyesters depending on their structures, e.g., chain lengths and substituents, has been systematically investigated in the vine-twining polymerization field. Furthermore, amylosic supramolecular network materials, such as hydrogels, are fabricated through vine-twining polymerization by using copolymers, where hydrophobic polyester guests or maltooligosaccharide primers are covalently modified on hydrophilic main-chain polymers. The vine-twining polymerization using such copolymers in the appropriate systems induces the formation of amylosic nanostructured inclusion complexes among them, which act as cross-linking points, giving rise to supramolecular networks at the nanoscale. The resulting materials form supramolecular hydrogels, films, and microparticles.

## 1. Introduction

**Figure 1:**
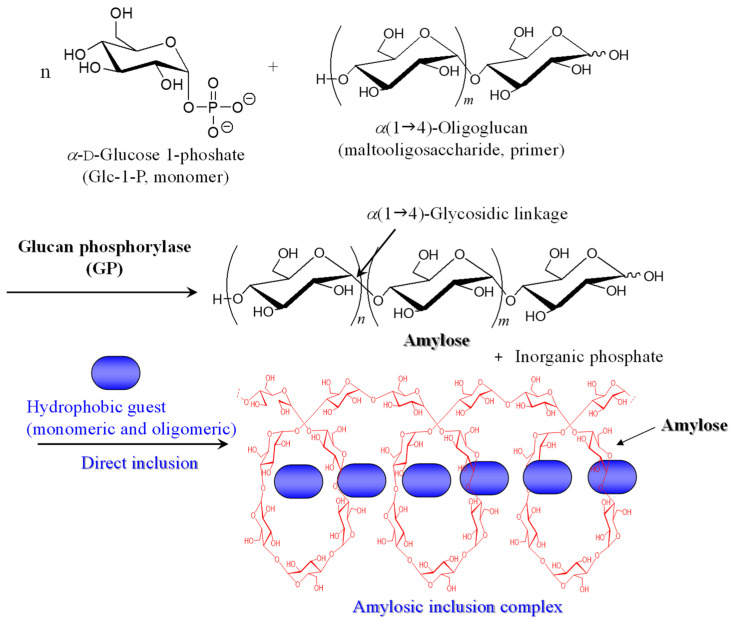
Glucan phosphorylase (GP)-catalyzed enzymatic polymerization of a-d-glucose 1-phosphate (Glc-1-P) as monomer using maltooligosaccharide as primer to produce amylose, which directly includes hydrophobic monomeric and oligomeric guests.

Polysaccharides are one of the representative biological macromolecules and act as important vital materials in nature [1,2,3]. Natural polysaccharides are typically composed of repeating monosaccharide units, linked through glycosidic bonds. Their higher-order and conformational nanostructures are regulated depending on the kinds of monosaccharide residues and glycosidic linkages, resulting in the specific function. For example, amylose, which is a natural polysaccharide as a component of starch [1], is known to show the following specific functions. Amylose, which comprises units of a(1g4)-linked d-glucose (Glc) with left-helical conformation, spontaneously forms a water-insoluble double-helical assembly [4,5]. Amylose is also a well-known host molecule, which directly includes hydrophobic guest substrates into its hydrophobic cavity through hydrophobic interaction to form amylosic nanostructured inclusion complexes (Figure 1) [6,7]. Suitable geometrical structures, typically monomeric and oligomeric sizes, are employed as guests to form amylosic inclusion complexes.

**Figure 2:**
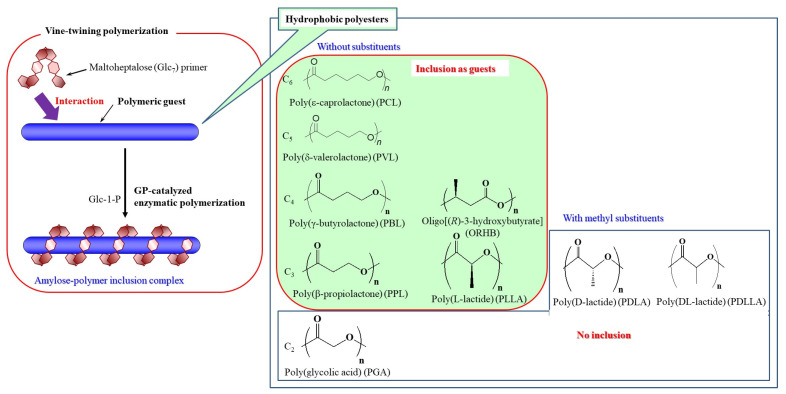
Image for vine-twining polymerization and hydrophobic polyesters, with amylose including or not including the vine-twining process.

Polymeric guests are potentially more favorable than small guests for such applications as functional supramolecular materials based on amylosic inclusion complexes because amylose–polymer inclusion complexes can be identified to show better qualities and properties than those of small molecules. However, only limited studies on the formation of such amylose–polymer inclusion complexes have been reported because of the difficulty in direct inclusion of polymeric long chains as guests into the amylosic cavity arising from the weak driving force, that is, hydrophobic interaction. As an example of the direct inclusion of polymeric guests by amylose, hydrophilic moieties have been attached at the chain ends of polyesters, which enhance the ability of amylose to helically include them in aqueous media [8,9]. The other methods for the formation of amylose–polymer inclusion complexes have been achieved through inclusion polymerization and guest-exchange approaches [10,11,12]. The direct mixing method under the selected conditions has also been investigated to form the inclusion complexes from amylose and polymeric guest molecules [13,14,15]. By means of dynamic elongation of the amylosic chain, the authors have developed a biomimetic method for the construction of amylose–polymer inclusion complexes [16,17,18], because polymeric assemblies are often constructed during a chain-elongation (polymerization) reaction in biological systems, e.g., formation of a double helix during DNA replication. Helical inclusion toward a polymeric chain during elongation from a shorter to longer amylosic chain occurs more readily than direct complexation of a polymeric guest with the amylosic cavity. The image of gradual helical inclusion toward the polymeric guest in an elongating amylosic cavity is similar to the way plant vines grow with twining around a support rod. Consequently, the above biomimetic approach for the formation of amylosic inclusion complexes with polymeric guests has been named ‘vine-twining polymerization’ (Figure 2) [19]. Besides amylose, b(1g3)-glucans, such as Schizophyllan, have also been found to form complexes with hydrophobic polymers [20,21,22].

The field of elongation of the amylosic chain is provided by glucan phosphorylase (GP)-catalyzed enzymatic polymerization, because the enzymatic approach has been known as an efficient tool for precisely synthesizing polysaccharides with regularly controlled structures [23,24,25]. GP-catalyzed enzymatic polymerization is carried out using a-d-glucose 1-phosphate (Glc-1-P) and maltooligosaccharide (a(1g4)-oligoglucan) as the monomer and primer, respectively (Figure 1) [26,27,28,29]. The initiation of the polymerization strictly occurs through the transfer of a Glc unit from Glc-1-P to the nonreducing end of the primer to produce the a(1g4)-linked Glc unit with the liberation of an inorganic phosphate. A propagation then takes place according to the consecutive transfer reactions at the elongating nonreducing end site of the amylosic saccharide chain. The GP-catalyzed enzymatic polymerization can be examined in the presence of appropriate hydrophobic polymeric guests dispersed in aqueous buffer as the polymerization medium. Consequently, the propagation proceeds with helical inclusion toward the polymeric chains by the amylosic cavity according to the vine-twining process to obtain amylose–polymer inclusion complexes (Figure 2) [19].

The first example of vine-twining polymerization was achieved using a hydrophobic polyether, that is, poly(tetrahydrofuran) (PTHF), as the polymeric guest to form an amylose-PTHF inclusion complex [30]. Since this finding, different kinds of hydrophobic polymers have been found to act as polymeric guests for the vine-twining polymerization to obtain the corresponding amylosic inclusion complexes. In particular, various hydrophobic polyesters have been employed to systematically investigate the helical inclusion behavior of amylose according to their structures (e.g., chain lengths, substituents) in the vine-twining polymerization field (Figure 2) [19]. After an overview is given of vine-twining polymerization, the present review article presents the helical inclusion behavior of amylose toward polyester guests through the vine-twining process, which is performed in the GP-catalyzed enzymatic polymerization field. The hierarchical nanoarchitecture of amylosic supramolecular networks composed of polyester guests is also disclosed, which has been achieved through vine-twining polymerization using polyester-grafted and primer-modified hydrophilic polymers. In these systems, amylosic inclusion complexes are formed among the hydrophilic polymeric chains, which act as cross-linking points to form amylosic supramolecular network hydrogels, films, and microparticles.

## 2. Overview of Vine-Twining Polymerization

As mentioned above, the author has found that helical inclusion toward hydrophobic polymeric guests by the amylosic cavity leads to the construction of inclusion complexes, biomimetically according to the vine-twining process in the following system. The GP-catalyzed enzymatic polymerization of the Glc-1-P monomer using the maltooligosaccharide primer, such as maltoheptaose (Glc_7_), is examined in the presence of suitable hydrophobic polymers, dispersed in aqueous buffer media (Figure 2) [19]. As the first example of vine-twining polymerization, the amylose-PTHF inclusion complex is obtained as a precipitate when GP-catalyzed enzymatic polymerization is carried out in aqueous buffer, where PTHF is dispersed [30]. The analytical results of the product suggested the inclusion complex structure. The following precise study on vine-twining polymerization using polyether guests has revealed a significance in the hydrophobicity of polymeric guests on the helical inclusion in the amylosic cavity [31]. Amylosic inclusion complexes are formed from hydrophobic polyethers, that is, PTHF and poly(oxetane) (POXT), whereas a hydrophilic polyether, that is, poly(ethylene glycol) (PEG), does not give rise to the formation of an amylosic inclusion complex under the same vine-twining polymerization conditions. When poly(oxepane) (POXP) with a stronger hydrophobicity than those of PTHF and POXT is used for the vine-twining polymerization, an inclusion complex is not obtained, because POXP does not exhibit dispersibility in aqueous buffer, which prevents its inclusion by the enzymatically elongated amylosic cavity. Moreover, vine-twining polymerization using poly(propylene glycol) (PPG), which is a structural isomer of POXT with methyl substituents, does not result in complexation by the amylosic cavity. The bulkiness of PPG compared to POXT probably prevents helical inclusion by the elongated amylosic chain during the GP-catalyzed enzymatic polymerization.

Based on the above results, the author concludes that the following characteristics are typically required for the polymeric guests that induce helical inclusion by amylose in the vine-twining polymerization system. Because the vine-twining polymerization is carried out in aqueous buffer as the solvent for the GP-catalyzed enzymatic polymerization, polymeric guests should contain relatively polar groups in their main chains for their dispersibility in such aqueous media. Polymeric guests should also exhibit moderate hydrophobicity for both dispersion in aqueous media and hydrophobic interaction with the amylosic cavity. Because a sufficient cavity size is not provided from amylose to include bulky polymeric gusts, slender polymers without bulky substituents can be conceived to be suitable guests for the vine-twining polymerization.

According to the above characteristics, poly(tetramethylene carbonate) (PTMC), with relatively polar carbonate groups in the main chain, that is, a hydrophobic polycarbonate, was found to act as a polymeric guest for the vine-twining polymerization to construct an amylosic inclusion complex [32]. Polycarbonates with a hydrophobicity stronger than PTMC (e.g., poly(dodecamethylene carbonate), poly(decamethylene carbonate), and poly(octamethylene carbonate)) have not induced the formation of amylosic inclusion complexes in GP-catalyzed enzymatic polymerization. Hydrophobic polyamides, that is, chiral polyalanine (PAlas) stereoisomers, were also used as polymeric guests for vine-twining polymerization [33]. In the three stereoisomers, i.e., poly(d-alanine) (PDAla), poly(l-alanine) (PLAla), and poly(dl-alanine) (PDLAla), only PDAla was helically included in the amylosic cavity in the field of GP-catalyzed polymerization. The helical directions of amylose and PAlas are key factors in explaining the stereoselective inclusion behavior in the vine-twining polymerization. Both amylose and PDAla construct a left-handed helical conformation, suitably giving rise to helical complexation between the two polymeric chains to obtain the amylosic inclusion complex.

Hydrophobic polyesters also provide suitable characteristics as polymeric guests for vine-twining polymerization. The author has extensively investigated the helical inclusion behavior of amylose toward such polyester guests depending on their structures. The following section deals with the significant findings in the vine-twining polymerization using hydrophobic polyester guests.

## 3. Vine-Twining Polymerization Using Hydrophobic Polyesters as Polymeric Guests

### 3.1. Slender Polyesters without Substituents as Polymeric Guests

Several hydrophobic slender polyesters without substituents, such as poly(ε-caprolactone) (PCL), poly(δ-valerolactone) (PVL), and poly(g-butyrolactone) (PBL), have been found to act as polymeric guests for vine-twining polymerization (Figure 2) [34,35,36]. When the GP-catalyzed enzymatic polymerization of Glc-1-P monomer was carried out using the Glc_7_ primer in aqueous buffer, which dispersed the polyester guests, amylose–polyester inclusion complexes were produced as precipitates. The characterization results of the precipitates fully supported the inclusion complex structures. In the same operation, a hydrophobic polyester with a shorter methylene chain than PBL was used, that is, poly(b-propiolactone) (PPL), and an incomplete inclusion of PPL in the amylosic cavity was observed. Furthermore, poly(glycolic acid) (PGA), which has an additionally shorter methylene chain, did not form the amylosic inclusion complex in the GP-catalyzed enzymatic polymerization in aqueous buffer, where a pure amylose was produced. The results of PPL and PGA were owed to the poor dispersibility of these polymers in aqueous media, as a result of their high crystallinities. When the copolyester, poly(glycolic acid-*co*-ε-caprolactone) (P(GA-*co*-CL)), was used as a polymeric guest, the corresponding amylosic inclusion complex was obtained using the vine-twining process in GP-catalyzed enzymatic polymerization. This was because of its higher dispersibility in aqueous media than that of the pure PGA, due to reduction in crystallinity by incorporating the PCL chains [37].

**Figure 3:**
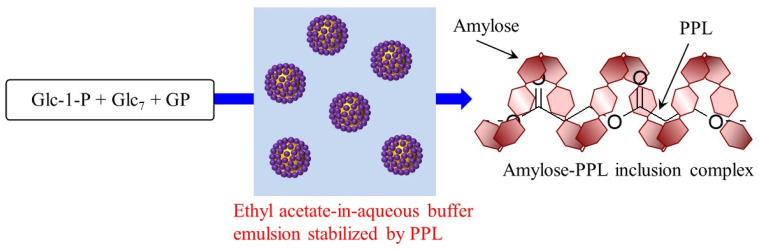
Vine-twining polymerization using PPL in ethyl acetate-in-aqueous buffer emulsion system to produce amylose-PPL inclusion complex.

To provide the dispersion of a pure PPL in aqueous media, an emulsion system was developed in the following study. The suspension of PPL in ethyl acetate solution, prepared through ultrasonication, was first mixed with an aqueous buffer. The obtained mixture was ultrasonicated to give rise to an emulsifying system, as the laser micrographic image of the produced system observed emulsified droplets. The image through laser micrographic measurement of a simple mixture of PPL with aqueous buffer after ultrasonication showed the morphology of microparticles, in addition to large aggregates. Such microparticles probably acted as stabilizers, giving rise to the formation of the emulsion system. When the GP-catalyzed enzymatic polymerization was conducted in the emulsion system containing dispersed PPL, helical inclusion of the enzymatically produced amylose toward PPL occurred, producing the amylose-PPL inclusion complex (Figure 3) [38]. It will be possible that PGA, which had not complexed with amylose through vine-twining polymerization also due to the poor dispersibility, could form of an inclusion complex by employing a similar emulsion system.

**Figure 4:**
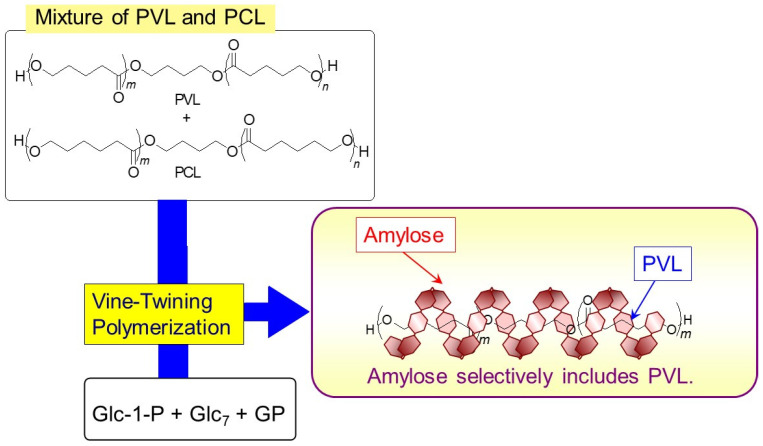
Selective inclusion of PVL by amylose in vine-twining polymerization system in the presence of PVL/PCL mixture.

A hydrophobic poly(ester-ether) composed of alternating ester and ether linkages (a unit structure; -CH_2_CH_2_C(C=O)OCH_2_CH_2_CH_2_CH_2_O-) also forms the amylosic inclusion complex through vine-twining polymerization [35]. Moreover, amylose has shown different inclusion behaviors in accordance with subtle changes in the structures of polymeric guests. For example, amylose selectively includes PVL to construct the inclusion complex through the vine-twining process when GP-catalyzed enzymatic polymerization is carried out in aqueous buffer, which disperses a mixture of PVL/PCL (Figure 4) [39]. The slight difference in the hydrophobicities of two polyesters probably caused the difference in the inclusion by amylose toward them. The hydrophobicity of PVL for helical inclusion by the amylosic cavity is more suitable than that of PCL.

### 3.2. Polyesters with Substituents as Polymeric Guests

Poly(lactic acid)s (PLAs) with methyl substituents, which are the structural isomers of PPL, have been employed as hydrophobic polyester guests for vine-twining polymerization. Three kinds of stereoisomers are present depending on stereo-arrangements of methyl substituents in PLAs, that is, poly(l-lactic acid) (PLLA), poly(d-lactic acid) (PDLA), and poly(dl-lactic acid) (PDLLA). In these stereoisomers, only PLLA forms an amylosic inclusion complex in the GP-catalyzed enzymatic polymerization field in aqueous buffer, which disperses PLAs (Figure 2) [40]. A key factor for whether PLAs form inclusion complexes with amylose or not is their helical directions, because PLLA constructs a heft-handed helix in the same manner as that of amylose, whereas PDLA and PDLLA construct opposite and irregular helical conformations. A similar phenomenon for helical inclusion by amylose depending on chirality was also observed in the investigation using PAlas as mentioned above [33].

Vine-twining polymerization was investigated using the structural isomer of PBL, which has substituents (methyl groups), that is, poly[(*R*)-3-hydroxybutyrate] (PRHB), as a guest polyester. As a result, only its oligomer with low molecular weight (approximately 500) gave rise to an amylosic inclusion complex under specific conditions in the GP-catalyzed enzymatic polymerization field as follows [41]. The enzymatic polymerization using oligo[(*R*)-3-hydroxybutyrate] (ORHB) is first examined at 70 °C, higher than that for the general vine-twining polymerization (at 45–50 °C), catalyzed by thermostable GP (from *Aquifex aeolicus* VF5), which shows stability at such a higher temperature. This polymerization system produces a water-soluble amylosic oligomer (short α(1γ4)-glucan) without double-helical assembly, called single amylose, that weakly interacts with ORHB. The polymerization mixture is then cooled to 45 °C over 7 h to carry out the further enzymatic propagation from the amylosic oligomer, catalyzed by GP, that helically includes ORHB to obtain the amylosic inclusion complex (Figure 2).

**Figure 5:**
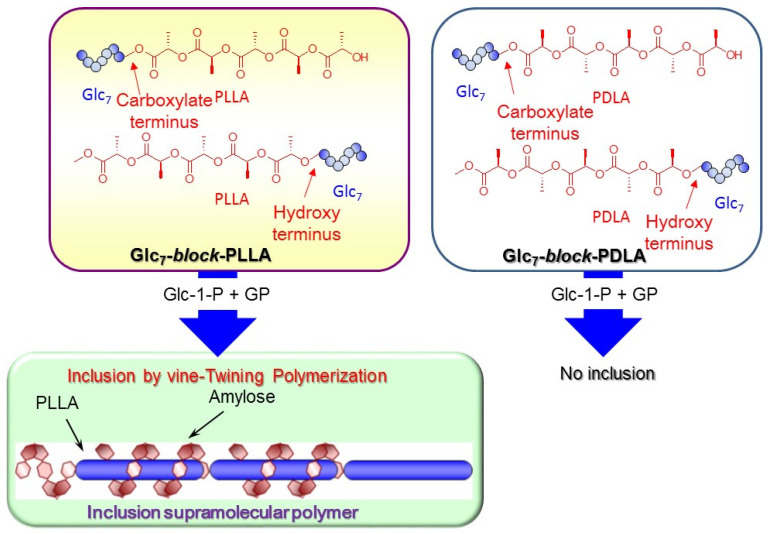
Primer–guest conjugates composed of PLLA from inclusion supramolecular polymers through vine-twining polymerization, whereas primer–guest conjugates composed of PDLA do not induce the formation of inclusion complexes.

The fabrication of an inclusion supramolecular polymer comprising amylose-PLLA inclusion complexes was achieved through vine-twining polymerizations using the designed guest polymers, i.e., Glc_7_-*block*-PLLA (a primer–guest conjugate) (Figure 5) [42]. This approach is based on the following strategic concept. An elongating amylosic chain initiated from the primer segment in the conjugate substrate through the GP-catalyzed enzymatic polymerization helically complexes with a guest polymer segment in another substrate. Consequently, such a helical inclusion among the substrates takes place, giving rise to the inclusion supramolecular polymer.

The relative orientation of the amylose and PLLA chains in the supramolecular polymer has been revealed using two Glc_7_–*block*-PLLA conjugates, which comprise a primer segment, linked to the carboxylate or hydroxy terminus of PLLA in the molecule. The GP-catalyzed enzymatic polymerization in the presence of both conjugates produces the inclusion supramolecular polymers according to the abovementioned process through the vine-twining polymerization process (Figure 5) [43]. These results suggest that regardless of the PLLA chain orientation, the enzymatically elongated amylose includes the PLLA segment. On the other hand, diblock copolymers comprising amylose–PDLA are produced in the GP-catalyzed enzymatic polymerization in the presence of the two Glc_7_-*block*-PDLA conjugates under the same conditions, but inclusion complexes are not formed (Figure 5). These results revealed that the helical inclusion behavior of amylose is strongly affected by PLLA chirality, irrespective of the PLA chain orientation. Helical inclusion complexation is induced by the left-handed helixes of both amylose and PLLA, while the methyl substituent orientation in PLLA, which is oppositely altered according to the relative chain orientation, does not affect inclusion complexation. This study has revealed that the helical direction of the guest polymer, which is responsibly induced by its chirality, strongly affects complexation with amylose regardless of the chain orientation.

**Figure 6:**
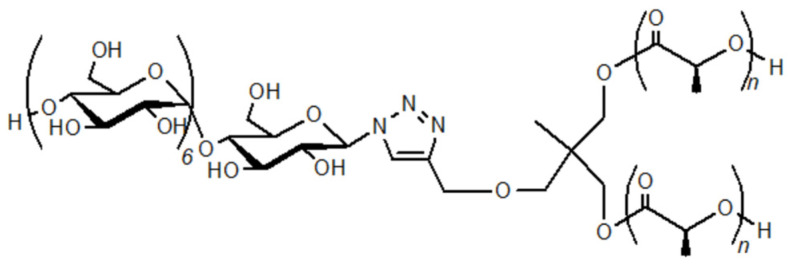
Structure of branched Glc_7_-PLLA (Glc_7_-PLLA_2_).

The synthesis of a hyperbranched supramolecular polymer composed of a continuum of amylose-PLLA inclusion complexes (amylose-PLLA_2_) was also carried out through the GP-catalyzed enzymatic polymerization using a branched Glc_7_-PLLA (Glc_7_-PLLA_2_) conjugate according to the vine-twining polymerization process (Figure 6) [44]. A supramolecular hydrogel of amylose-PLLA_2_ was obtained through the formation of an ion gel with an ionic liquid, 1-butyl-3-methylimidazolium chloride (BMIMCl). Furthermore, a cryogel with porous morphology was produced through lyophilization of the hydrogel.

## 4. Hierarchical Nanoarchitecture of Amylosic Supramolecular Network Materials Consisting of Polyester Components through Vine-Twining Polymerization Approach

The vine-twining polymerization process for the formation of inclusion complexes with polyester guests has been applied to the hierarchical nanoarchitecture of amylosic network materials, which appropriately form supramolecular materials, such as hydrogels and films [45]. Cross-linking points are obtained from the inclusion complexes, constructed among side-chains on suitable polymeric main chains to fabricate supramolecular network structures at the nanoscale. Two types of copolymers have been designed, where polyester guests are covalently linked on polymeric main chains and the reducing ends of maltooligosaccharide primers are modified on polymeric main chains. When the former graft copolymers are used, elongated through the GP-catalyzed enzymatic polymerization, the grafted polyester guests are helically included to form amylosic inclusion complexes among graft copolymers, which act as cross-linking points to construct the supramolecular networks at the nanoscale.

**Figure 7:**
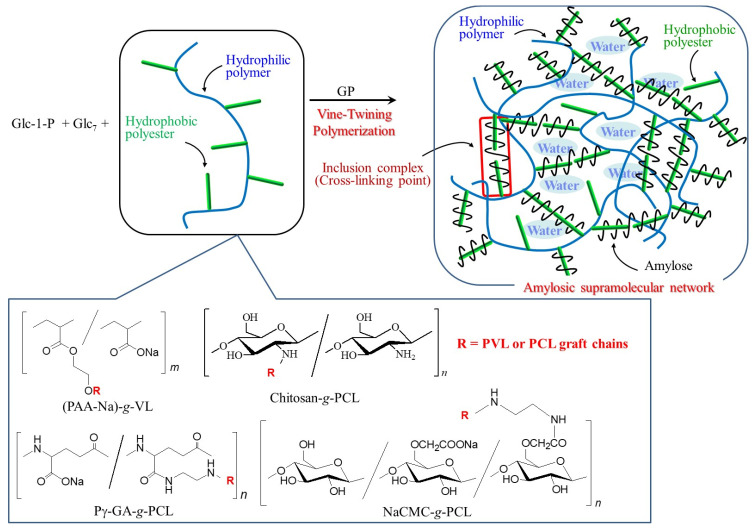
Fabrication of amylosic supramolecular networks through vine-twining polymerization using graft copolymers having hydrophilic main chains and hydrophobic polyester graft chains.

For example, poly(acrylic acid sodium salt-*graft*-δ-valerolactone) ((PAA-Na)-*g*-PVL) was used. When GP-catalyzed enzymatic polymerization is examined in the presence of (PAA-Na)-*g*-PVL in aqueous buffer, the reaction mixture completely turns into the hydrogel form [46]. The characterization results of the hydrogel support the assumption that inclusion complexes are obtained by amylose with the intermolecular ((PAA-Na)-*g*-PVL)s through the vine-twining polymerization process, which act as cross-linking points for the construction of the amylosic supramolecular network at the nanoscale (Figure 7). The hydrogel is disrupted and reproduced through the β-amylase-catalyzed hydrolysis of amylose and the reformation of amylose through GP-catalyzed polymerization, respectively.

Chitosan-*graft*-poly(ε-caprolactone) (chitosan-*g*-PCL) has also been used for the same approach to fabricate the amylosic supramolecular network at the nanoscale (Figure 7) [47]. Because the hydrogel produced entirely comprises biodegradable components, that is, amylose, PCL, and chitosan, it can be disrupted through the enzymatic hydrolysis of respective components, catalyzed by β-amylase, lipase, and chitosanase. When the GP-catalyzed enzymatic polymerization is performed in the presence of another graft copolymer, poly(γ-glutamic acid)-*graft*-poly(ε-caprolactone) (Pg-GA-*g*-PCL) (Figure 7), a self-standing hydrogel is obtained [48]. Its mechanical properties are superior to those of the supramolecular hydrogels, formed using PAA-Na-*g*-PVL and chitosan-*g*-PCL. Macroscopic interfacial healing of the resulting hydrogel comprising Pg-GA-*g*-PCL is achieved through GP-catalyzed enzymatic polymerization. The hydrogel is cut into two portions, and then the GP-catalyzed enzymatic polymerization solution (containing Glc-1-P monomer and Glc_7_ primer) in aqueous buffer is placed on the surfaces of the portions. After the surfaces contact with each other, the resulting material is kept stable under the GP-catalyzed enzymatic polymerization conditions. Consequently, the two hydrogel portions are healed at the contacted point. The healing of the hydrogel on a macroscopic level is successfully progressed through the formation of the amylosic inclusion complex from the enzymatically elongated amyloses with the PCL graft chains at the interface.

**Figure 8:**
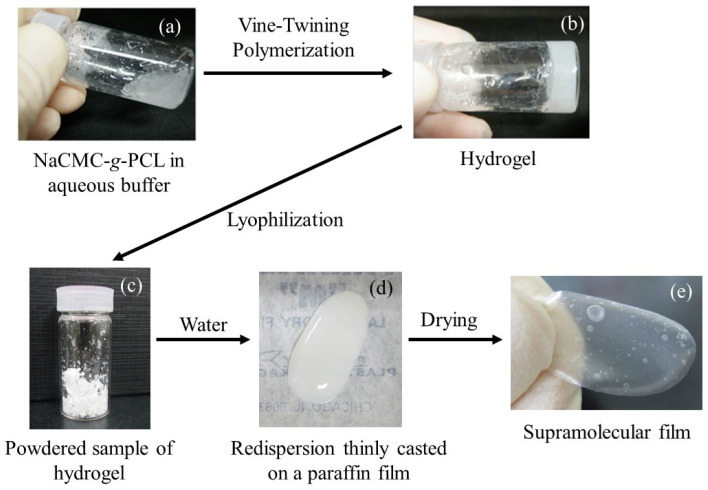
Photographs before (**a**) and after (**b**) vine-twining polymerization using NaCMC-*g*-PCL, powdered sample (**c**), redispersion (**d**), and supramolecular film (**e**) (adapted with permission from Ref. [49]. Copyright 2013, Elsevier).

A supramolecular film is obtained via the hierarchical construction of a supramolecular hydrogel through vine-twining polymerization in the presence of carboxymethyl cellulose-*graft*-poly(ε-caprolactone) (NaCMC-*g*-PCL) (Figure 7) [49]. The reaction mixture in the GP-catalyzed enzymatic polymerization in the presence of NaCMC-g-PCL was totally transformed into a hydrogel through the formation of the amylosic supramolecular network at the nanoscale (Figure 8). The gel was then converted into the supramolecular film by moisturizing a lyophilized sample from the supramolecular hydrogel according to the procedure shown in Figure 8. The present films are expected to be used in practical applications as eco-friendly and functional bio-based materials in the future.

**Figure 9:**
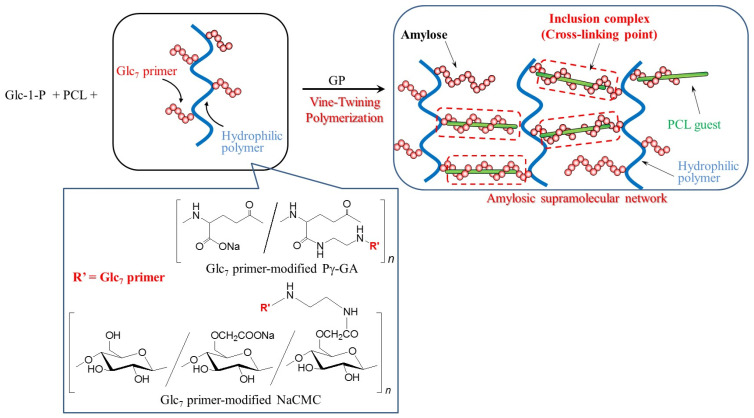
Fabrication of amylosic supramolecular networks through vine-twining polymerization using Glc_7_-primer-modified Pg-GA and NaCMC in the presence of PCL.

**Figure 10:**
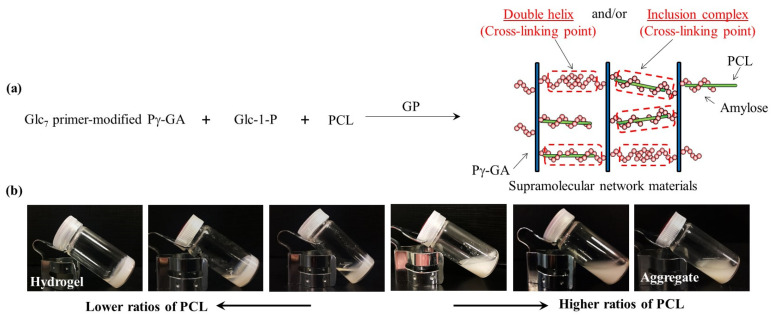
(**a**) Image for preparation of supramolecular network materials by means of amylose helical assemblies formed via GP-catalyzed enzymatic polymerization and (**b**) photographs of reaction mixtures (**b**) (adapted with permission from Ref. [50]. Copyright 2018, Elsevier).

Another approach to construct amylosic supramolecular networks through the vine-twining polymerization process has been investigated through the GP-catalyzed enzymatic polymerization in the presence of PCL using Glc_7_ primer-modified Pg-GA and NaCMC (Figure 9) [50,51,52]. PCL/primer feed ratios strongly affect the macroscopic structures of the resulting amylosic supramolecular networks as shown in Figure 10. Lower ratios of PCL give rise to the hierarchical nanoarchitecture of larger networks, mostly composed of amylosic double helixes, leading to the formation of supramolecular hydrogels from the reaction mixtures. In contrast, the presence of higher ratios of PCL induces smaller nanonetworks to form, largely comprising the amylosic inclusion complexes with PCL, according to the vine-twining polymerization process, giving rise to aggregates in the reaction mixtures.

**Figure 11:**
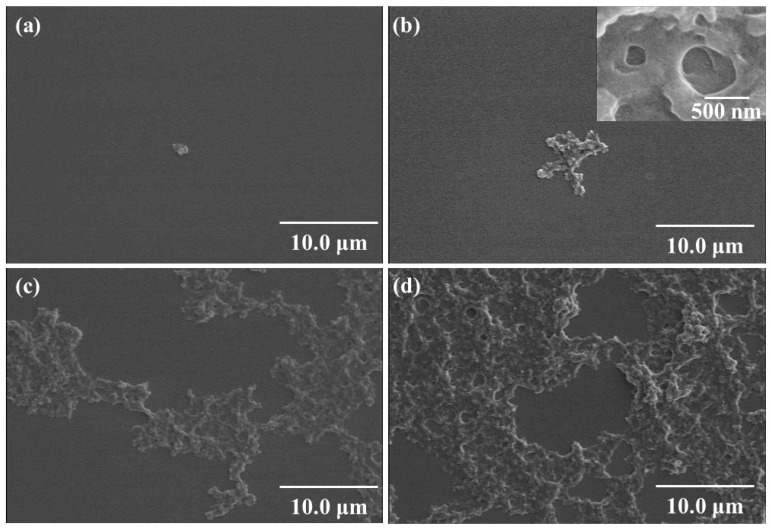
SEM images of spin-coated samples from reaction mixtures of vine-twining polymerization using Glc_7_-primer-modified Pg-GA in the presence of PCL (reaction times = 3 (**a**), 8 (**b**), 15 (**c**), and 21 h (**d**) (reprinted with permission from Ref. [50]. Copyright 2018, Elsevier).

To evaluate the formation process of amylosic supramolecular networks via vine-twining polymerization using Glc_7_-primer-modified Pg-GA in the presence of PCL, the samples, lyophilized from reaction mixtures according to increase in reaction times, were subjected to SEM measurement (Figure 11). The SEM image of the sample from the reaction time of 3 h shows the morphology of the small pore (Figure 11a). Besides the smaller pores, the production of the larger pores with longer reaction times was recognized in the SEM images of the samples (Figure 11b–d). A smaller network was constructed on the molecular level by inclusion complexes of the short amylose graft chains with the PCL-formed inclusion complex with PCL according to the vine-twining polymerization process, which then aggregated to fabricate the smaller pore morphology at the macroscopic level. In the latter stage, the longer amylose chains gradually formed a larger network with double-helix cross-linking points, which accordingly constructed the larger pore morphology.

The fabrication of macroscopic structures from amylose-grafted Pg-GA networks was attempted [53]. Because the enzymatic polymerization using thermostable GP (from *Aquifex aeolicus* VF5) at elevated temperatures has been found to produce a water-soluble amylosic product with a single chain (called single amylose) in an equilibrium state between polymerization and phosphorolysis (chain elongation and cleavage reactions, respectively), the enzymatic polymerization from the primer-modified Pg-GA was first conducted with or without PLLA at 80 °C for single-amylose-chain production [41]. Double-helix formation was then performed by cooling the reaction mixture at room temperature. Accordingly, the following formation processes of the microparticles from the resulting amylose-grafted Pg-GA networks were proposed. During this process without PLLA, intermolecular networks among the graft copolymers are fabricated through double-helix formation from the enzymatically produced single amylose graft chains among the Pg-GA main chains. By further cooling, the growth of stable spherical morphologies took place through the additional production of the double helixes from the single amyloses among the networks, resulting in the microparticles. A similar process for microparticle formation is also conceived in the systems with PLLA to obtain the single amylose graft chains, but which do not result in inclusion complexes. By cooling after the enzymatic reaction, inclusion complex formation from a part of the single amylose graft chains with PLLA among the Pg-GA main chains initially gave rise to the network assemblies as nuclei of the particles. The microparticles were then fabricated through the following double-helix formation from the rest of the amylose graft chains among the nuclei. The increase in average diameters according to the PLLA/primer feed ratios was confirmed through the dynamic light scattering measurement of the mixtures by cooling for 1 h after the enzymatic polymerization at 80 °C. The results indicated that the larger nuclei were fabricated through initially assembling the larger numbers of the amylose-grafted Pg-GAs via inclusion complexation with PLLA according to the feed ratios obtained. Therefore, the larger microparticle formation was strongly affected by the sizes of the nuclei. The resulting microparticles comprising the amylose-grafted Pg-GA networks will have the potential to be practically applied as new bio-based functional materials with regularly controlled micromorphology.

## 5. Conclusions

The present review comprehensively overviewed the helical inclusion of amylose toward various hydrophobic polyester guests in the biomimetic vine-twining polymerization process. The investigation examined, regarding GP-catalyzed enzymatic polymerization, whether amylosic inclusion complexes form from the hydrophobic polyesters depending on their structures. Polyesters, such as PCL, PVL, and PBL, which exhibit dispersion ability in aqueous polymerization media, are helically included in the amylosic cavity via the vine-twining polymerization process. The highly crystalline polyesters, that is, PPL and PGA, show low dispersibility in aqueous media, leading to difficulty in the progress of vine-twining polymerization. Dispersion in the emulsion system and a reduction in the crystallinity by incorporating the other polyester in the main chain from PPL and PGA, respectively, induce the vine-twining process in the GP-catalyzed enzymatic polymerization. The helical directions of the polyesters with methyl substituents depending on chirality strongly affect the helical inclusion by the amylosic cavity in the vine-twining polymerization process. Amylosic supramolecular networks at the nanoscale are hierarchically fabricated through the construction of cross-linking points from the amylose–polyester inclusion complexes in the vine-twining process in the GP-catalyzed enzymatic polymerization using polyester-grafted and primer-modified hydrophilic polymers. The products formed supramolecular materials, such as hydrogels, films, and microparticles. A variety of polyester structures are available via simple ring-opening polymerization of the corresponding cyclic monomers, compared with other hydrophobic polymers containing polar groups, such as polyethers and polycarbonate. Accordingly, a systematic study on the vine-twining polymerization using the polyester guests has previously been investigated, as mentioned above. In the future, new hydrophobic polyesters will be found to act as guests for vine-twining polymerization, which could also be potentially used for the fabrication of amylosic supramolecular network nanomaterials, like gels, with unique functions. In addition, the recycling and reuse of GP are key techniques for the scale-up of the present amylosic supramolecular network materials in practical applications. Such procedures will be developed by employing enzymological technology in the GP-catalyzed enzymatic polymerization.

## Data Availability

Not applicable.
